# A fast, easy circumcision procedure combining a CO2 laser and cyanoacrylate adhesive: a non-randomized comparative trial

**DOI:** 10.1590/S1677-5538.IBJU.2015.0284

**Published:** 2016

**Authors:** Tahsin Gorgulu, Abdulkerim Olgun, Merve Torun, Eksal Kargi

**Affiliations:** 1 Department of Plastic, Reconstructive and Aesthetic Surgery, Bulent Ecevit University Medical Faculty, Zonguldak, Turkey

**Keywords:** Circumcision, Male, Cyanoacrylates, Lasers

## Abstract

**Background:**

Circumcision is performed as a routine operation in many countries, more commonly for religious and cultural reasons than for indicated conditions, such as phimosis and balanitis. There are many techniques available, and recently electrocautery and both Nd:YAG and CO_2_ lasers, instead of blades, have been used for skin and mucosal incisions. However, the infection risk in circumcisions performed using a CO_2_ laser was 10% higher. There are also reports of sutureless procedures using cyanoacrylate, but these have higher risks of hematoma and hemorrhage. We combined a CO_2_ laser and cyanoacrylate to shorten the operation time and to decrease bleeding complications.

**Materials and Methods:**

: Circumcisions were performed under general anesthesia with CO2 laser and cyanoacrylate combination in 75 6–9-year-old boys between May 2013 and August 2014 only for religious reasons. As a control, we compared them retrospectively with 75 age-matched patients who were circumcised using the conventional guillotine method in our clinic.

**Results:**

No hematomas, bleeding, or wound infections were observed. One wound dehiscence (1.33%) occurred during the early postoperative period and healed without any additional procedures. The median operating time was 7 (range 6–9) minutes. The conventional guillotine group comprised one hematoma (1.3%), two wound dehiscences (2.6%), and two hemorrhages (2.6%), and the median operating time was 22 (range 20–26) minutes. The difference in surgical time was significant (p<0.001), with no significant difference in the rate of complications between the two groups.

**Conclusion:**

The combined CO2 laser and cyanoacrylate procedure not only decreased the operating time markedly, but also eliminated the disadvantages associated with each individual procedure alone.

## INTRODUCTION

Circumcision, which is performed more commonly for religious and cultural reasons than for indicated conditions, such as phimosis and balanitis, is a routine operation in many countries and has a centuries-long history (1). Currently, it is the most common surgical procedure performed in male children (2). Recent studies have shown that it increases penile hygiene and decreases the risks of penile cancer and human immunodeficiency virus (HIV) infection (3-6).

Surgically, circumcision is an easy technique to learn and perform. However, the results must be satisfactory both functionally and esthetically. There are many techniques for performing circumcision. In recent years, circumcision procedures using auxiliary devices have become popular (7, 8). However, the use of these devices increases the recovery time (9).

The guillotine technique is a simple, quick method for circumcision. This technique involves excising the preputial skin circumferentially, preserving the glans penis after separating the adhesions of the coronal sulcus. To preserve the glans penis, the preputial skin is pulled up, and a straight hemostat is applied loosely to the preputium. The excess skin is cut using a scalpel between the glans and hemostat. However, this method has many shortcomings (10).

Electrocautery and Nd:YAG and CO_2_ lasers are frequently used for circumcision, instead of blades for the skin and mucosal excisions (11-13). CO_2_ lasers have been used since the early 1970s and are widely used for dermatology and plastic surgery procedures (14, 15). Many clinics also routinely use tissue adhesives to treat facial incisions (16). There are some reports on sutureless circumcision using cyanoacrylate (17, 18). However, very recently only one report has used the combination of a CO_2_ laser and cyanoacrylate for circumcisions in a small series (30 patients) (19). Therefore, this study examined the combined use of a CO_2_ laser and cyanoacrylate for shortening the operating time and reducing complications related to bleeding, in comparison with the conventional guillotine method in a bigger series with 150 patients.

## MATERIALS AND METHODS

**Figure 1:**
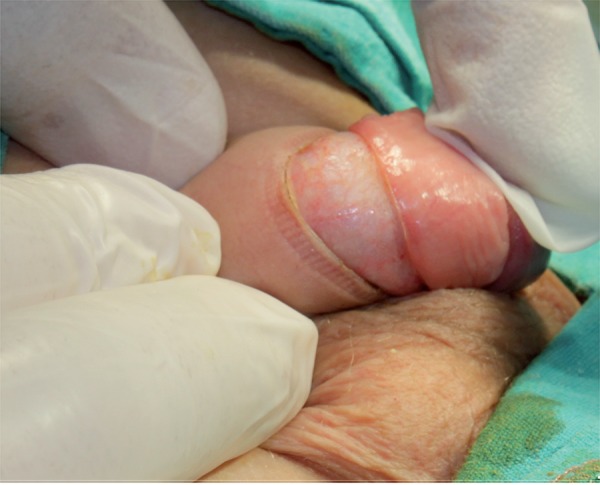
Image obtained after laser excision.

**Figure 2:**
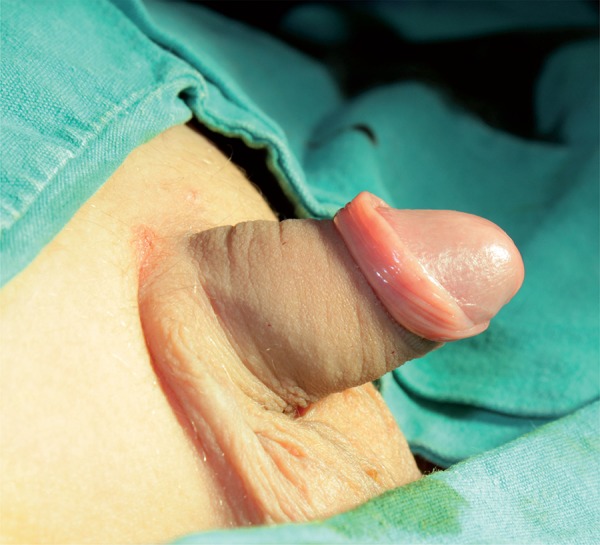
Perioperative image obtained after cyanoacrylate application.

We performed our technique on 75 boys, aged 6–9 (median 7) years, who underwent circumcision between May 2013 and August 2014. In all cases, the parents had requested circumcision for religious reasons. Routinely, after iodine disinfection under general anesthesia, the adhesions on the coronal sulcus are separated, the preputial skin is pulled up, and a straight hemostat is applied loosely to the preputium. The incision is made distal to the hemostat using a CO_2_ laser (UltraPulse 5000C, Coherent Medical Group, Santa Clara, USA), applying 350 millijoules energy at 40 pulses/second in continuous mode. No bleeding requiring bipolar use was detected after the excision (Figure-1). Cyanoacrylate (Leukosan Adhesive, BSN Medical, Hamburg, Germany) is applied after approximating the mucosa and remaining skin on the penis (Figure-2). A chlorhexidine wound covering and Coban bandage are used for wound care.

The complications and operating time in this group were compared retrospectively with those of 75 age-matched patients from our clinic archive who were circumcised using the conventional guillotine method for religious reasons (supplementary material Video-1).

## RESULTS

**Figure 3:**
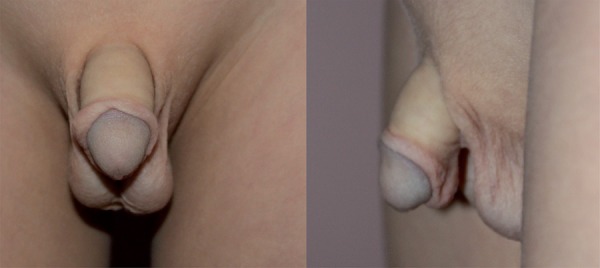
Anterior and lateral images obtained during the 6th month.

The patients were followed postoperatively for 12 (range 4–18) months on average. Wound healing took one week. No hematomas, bleeding, or wound infections were observed. Dehiscence occurred in one child (1.3%) during the early postoperative period but healed spontaneously within one week. Six months postoperatively, the cases were similar in appearance to those who underwent the conventional procedure (Figure-3). The parents of the patients were satisfied with the aesthetic results. The median operating time was 7 (range 6–9) minutes. In the conventional guillotine group, one hematoma (1.3%), two wound dehiscences (2.6%), and two hemorrhages (2.6%) were recorded, and the median operating time was 22 (20–26) minutes. The difference in operating times between the groups was significant (p<0.001), while the difference in complications was not (p>0.5) (Table-1).

**Table 1:** Comparison of conventional guillotine group and laser+cyanoacrylate group.

	Conventional Guillotine Group (75 Patients)	Laser + Cyanoacrylate Group (75 Patients)	p Value
Operation Time (Median)	22 minutes	7 minutes	***p* <0.001**
Hematoma	1(%1.3)	0	p>0.5
Wound Dehiscence	2(%2.6)	1(%1.3)	p>0.5
Hemorrhage	2(%2.6)	0	p>0.5

## DISCUSSION

Circumcision is performed routinely in many countries for religious and cultural reasons. Generally, it is performed in pediatric patients and protects against penile cancer and HIV (3-6).

Commonly used conventional circumcision techniques include the dorsal slit, sleeve, and guillotine techniques (20). Electrocautery or Nd:YAG or CO_2_ lasers can be used instead of a scalpel. In addition, non-invasive circumcision using various devices has become popular (7, 8), although the recovery period is increased when these devices are used (9).

Procedures performed using a CO_2_ laser can shorten the operating time and decrease bleeding and pain (21). How et al. (13) compared the costs associated with the operating time between CO_2_ lasers and the conventional technique. They found that the median operating time was 20 (range 16–21) minutes using the conventional technique and 15 (range 13–17) minutes using a CO_2_ laser. When all of the expenses accrued were calculated, the CO_2_ laser technique was much more cost effective for circumcision than was the conventional technique, and the morbidity rates were favorable compared with the conventional technique. We could not perform a cost-analysis, because the operating theatre charges in our university hospital are not known due to the health policies in our country. Nevertheless, our median operating time was less than half that of How et al., suggesting that our technique is more cost-effective.

Although the postoperative complication risks of circumcision performed using a scalpel versus a CO_2_ laser were similar, the infection risk was 10% higher with the CO_2_ laser (10, 22-23).

Recently, the use of tissue adhesives has increased, because they are easy to use, shorten the procedure duration, and achieve similar cosmetic results to those using standard suturing techniques (25-27). Antimicrobial effects, especially on Gram-positive bacteria, are very important for wound care (28). Another important advantage of tissue adhesives is that they decrease the risks of granulation tissue and sinus tract formation (17).

When compared with conventional techniques, circumcision using a CO_2_ laser has many advantages, while the increased risk of postoperative infections is the main disadvantage. The antimicrobial characteristics of tissue adhesives overcome this problem associated with laser circumcision. On the other hand, tissue adhesives carry risks of hematoma formation and related suture detachment. However, the high level of hemostasis in circumcisions performed using a CO_2_ laser prevents hematoma development and any related detachment.

Complications such as hematoma and hemorrhage, which Kelly et al. (17) reported in 502 subjects undergoing sutureless circumcision, were not encountered using the cyanoacrylate and laser combination in our study. Our complication rates were 1.4% for hematomas and 2.2% for hemorrhage using standard treatment and 0% in the combination group. The dehiscence rate was similar to that in our study (0.8%).

We did not evaluate postoperative pain in this study. Furthermore, a very recently report shown that pain score is not significant different between laser and conventional technique (19).

The parents of the patients in both groups were satisfied with the aesthetic results in our study.

The main reason we developed the combination technique was to prevent complications such as hematoma and hemorrhage by using the laser, while shortening the operation time, decreasing the infection risk, and eliminating the need for postoperative suture removal, which is difficult to perform in children, by using cyanoacrylate.

In the light of our results, we believe that our combination method overcomes the disadvantages observed with each circumcision procedure alone and shortening the operating time.

## Supplementary Material

**Video 1 -** Conventional Guillotine Technique and Guillotine incision with CO_2_ Laser.

Link: http://www.intbrazjurol.com.br/videos/IBJU-2015-0284video.flv

## Notes

**Video 1 -** Conventional Guillotine Technique and Guillotine incision with CO_2_ Laser.

Link: http://www.intbrazjurol.com.br/videos/IBJU-2015-0284video.flv
